# Public Events and Flashbulb Memories in Parkinson’s Disease

**DOI:** 10.1155/2008/389181

**Published:** 2008-04-11

**Authors:** Céline Borg, Catherine Thomas Antérion, Hélène Vioux, Aurélia Poujois, Bernard Laurent

**Affiliations:** ^1^NeuropsychologyCMRR UnitCHU Bellevue42055 Saint Etienne cx 02France; ^2^Neurology DepartmentCHU Bellevue42055 Saint Etienne cx 02France

**Keywords:** Parkinson’s disease, public events memory, flashbulb memory, executive functions, remote memory, anterograde memory

## Abstract

Public events and Flashbulb memories were investigated in 12 non-demented patients with Parkinson’s disease (PD) and 12 controls. Knowledge of public events and flashbulbs memories were assessed using a Famous Events Test (EVE 30). Contributions of semantic, episodic, as well as executive functioning and anterograde memory were examined. Results primarily showed that the performances of patients with PD were lower than these of controls in 4 tasks: free recall, specific questions, dating events and date recognition. They also had difficulties in finding the temporal order of 8 events. In contrast, the PD group benefited from events recognition themselves to the same extent as the controls. Secondly, the recall of flashbulb memories (FBM) was lower in the PD group than in the controls. Finally, correlations appeared in PD between the detailed recall of the events with the “recall” abilities of the MATTIS scale, possibly reflect an impairment in rebuilding memories. A positive correlation is also observed with the initiation score of the MATTIS (executive component), suggesting that the difficulties of rebuilding can be related to a dysfunction in accessing information because of a certain degree of frontal amnesia.

## 1. Introduction

Public events memory generally refers to semantic knowledge but, we are also able to date an event [1] and to retain vivid and detailed recollections of it. This last phenomenon is called "Flashbulb Memory" (FBM). The hallmark of FBM is that individuals are able to remember the specifics of the context in which they first heard of a public event (autobiographical episodic memory). It is linked to an emotional and surprising dimension which partly allows its long-term creation and preservation [2,3]. FBM would be impaired in older adults [4–6], reflecting an episodic source deficit [6] with reduced frontal lobe function [7,8]. Even though some studies have shown no relation between the frontal ability and the recall of FBM [9], the frontal lobes appear to play a specific role in strategic processes associated with the organization and monitoring of information at the moment of encoding and retrieval.

In Parkinson's disease (PD), the degeneration of the nigrostriatal dopaminergic system involves a disruption of the striato-frontal circuits constituting the subcortical-cortico-frontal syndrome. The executive functions are modified very early in the disease [10], in particular the planning, shifting and organisation of time [11]. Some studies have provided evidence for normal performances in recall of the content of public events but impaired abilities in dating capacities [12, 13], while others [14] have shown that the control group significantly recalled, dated and recognized the date better than the PD group. A temporal gradient was reported for PD patients with dementia [14,15] for the most recent decades [14], but this gradient is not always found.

The aim of the current study is to examine several simultaneously occurring events that are likely to gen-

ISSN 0953-4180/08/$17.00 © 2008 – IOS Press and the authors. All rights reserved erate a FBM on normal aging and non-demented PD patients. To the best of our knowledge, no research has investigated the recall of both public events and FBM on PD patients. The frontal dysfunction in PD, in terms of processes of control, judgement of plausibility and source memory [16] allows us to predict difficulties in the public events memories rebuilding by considering the semantic and episodic dimensions, and to explain certain complaints by patients who have the feeling that they are less able to record memories.

## 2. Method

### *2.1. Participants*

Twelve non-demented PD patients and 12 healthy controls matched for age, education and sex, participated in this study. Nine of the PD patients were treated with dopatherapy. The mean duration of the pathology was five years. Patients had no cognitive impairment interfering with their autonomy. Patients underwent the dementia rating scale of MATTIS (1976), including executive functions and anterograde memory processes. Demographic characteristics of the participants are presented in Table 1.

Table 1
Participant demographics

| | PD patients ($n$ = 12) | Controls ($n$ = 12) |
|---|---|---|
| Age | 72 (± 4,16) | 72,08 (± 4,62) |
| Sex ratio M/F | 7/5 | 7/5 |
| Education level | 12,64 (± 1,50) | 12,42 (±1,20) |
| MMSE score | 29 (± 0,85) | 29,41 (± 0,66) |
| Total MATTIS score | 138,8 (± 4,63) | |
| Attention MATTIS score | 35,71 (± 1,11) | |
| Initiation MATTIS score | 38,85 (± 2,5) | |
| Concept MATTIS score | 24 (± 1,41) | |
| Recall MATTIS score | 6,28 (± 1,11) | |

Note. MMSE (Mini-Mental State Examination (Folstein, Folstein & Mc Hugh, 1975); MATTIS (Dementia rating scale of MATTIS, 1976)).

### *2.2. Material and instructions*

We used 27 public events in the Famous Events Test, called EVE 30. This test was normalized on a normal French population and was based on events which occurred between 1939 and 2005. All the events were contemporary with the subjects [17,18] and were presented verbally. The experimental tasks included the following steps: free recall (responses were awarded 0, 1, or 2 points based on accuracy), recognition (responses were quoted 0 or 0.5 point), specific questions (participants had to answer two specific questions related to the corresponding event; 0 or 1 point was allocated per question), dating events (1 point per each defined decade) and date recognition (0 or 0.5 point).

Secondly, participants had to recall the personal circumstances in which they had first heard of an event (e.g., where, how, at what time, with whom?) Each correct response was scored 1.5. The maximum score for FBM was 6.

Finally, at the end of this test, we added a chronological classification test of 8 of the 27 events (one per period). Their order of presentation was randomized.

## 3. Results

Two participants (one control and one PD patient) were excluded from the analyses.

### *3.1. Public events memories*

A 3-factors ANOVA (group, task, time period), with a repeated measure on task and time period, showed a significant interaction between group and task (F(24,480) = 6.63, $p = 0.0001$) (see Fig. 1).

For between comparisons, controls performed better than the PD group in free recall (t (20) = -4.58, $p = 0.0002$), specific questions (t (20) = - 2.01, $p = 0.05$), dating events (t (20) = -3.85, $p = 0.001$) and date recognition (t (20) = -6.12, $p < 0.0001$). In contrast, controls and patients with PD showed the same ability to recognize the events. No interaction between group and time period appeared.

### *3.2. Temporal order*

It was also shown that patients with PD were impaired in their ability to find the chronological order of the 8 events, in comparison with normal controls (t (20) = $-4.18$, $p = 0.0005$).

### *3.3. Flashbulb memories*

Statistical analysis of the data recorded on the FBM was also processed and revealed that patients with PD had less FBM (23%) than controls (24%) (F(1.20) = 4.21, $p = 0.05$). The group variable did not interact with time period.

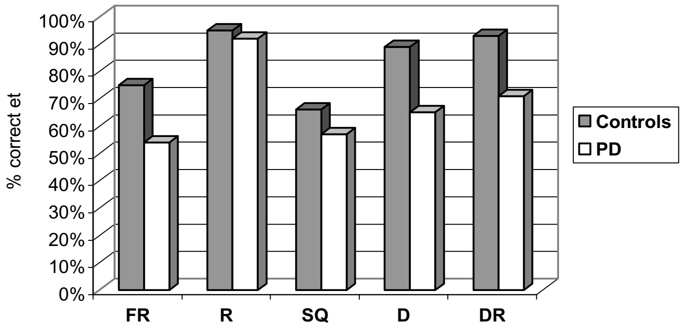

Fig. 1. Overall performance of patients with PD and normal controls on the Famous Events Test showing mean scores in percentage for free recall, recognition, specific questions, dating and date recognition conditions.

### *3.4. About the link between FBM and public events memories*

We assumed that events associated with a FBM should be better recalled than events not associated with a FBM. Therefore, we considered the events very closely associated with a FBM (score $\geqslant 3$) and the ones less associated (score $\leqslant$3). The pattern of results revealed that the mean public events memory scores associated with a FBM recall (mean score: 4.59) were significantly higher than public events memory scores which were not associated with a FBM recall (mean score: 3.98), F(1.20) = 38.15, $p = 0.0001$. This effect is significant for both groups.

### *3.5. Spearman correlations*

Spearman correlations were conducted for the patients with PD. Positive correlations were revealed between the "recall of two phrases" MATTIS scale and the total Famous Events Test score ($r = 0.78$; $p = 0.04$), the specific questions score ($r = 0.78$; $p = 0.04$) and the FBM score ($r = 0.77$; $p = 0.04$) and between the "memory" MATTIS scale and the total Famous Events Test score ($r = 0.77$; $p = 0.04$). Finally, two positive correlations between the "initiation" MATTIS score and the total Famous Events Test ($r = 0.75$; $p = 0.04$) and the specific questions scores ($r = 0.93$; $p = 0.007$) also appeared.

## 4. Discussion

It is worth pointing out that patients with PD performed relatively well in the Famous Events Test (68% of responses were correct), better than patients with Alzheimer's disease or MCI [19]. Nevertheless, patients with PD were impaired in recalling the content of public events: they had difficulty in evoking an event and in answering the specific questions. By contrast, the PD group did not differ from the control in the recognition task, possibly reflecting an access problem [12,14]. Furthermore, they showed a deficit in dating events, recognizing dates and finding the temporal order, suggesting a contextual deficit but also a deficit of the executive functions [20].

The present results about the content of events are not consistent with the data observed by Fama et al. (2000) [12]. There were differences between the methodology used by these authors and ours. First, the age and the MMSE score were quite different. Secondly, the paradigm differs in its nature and characteristics. Our paradigm necessitated a more important field of knowledge (about 27 events with a precise spatiotemporal context located in various fields versus candidates in American presidential elections in the Fama et al. study). To identify the political engagement of a candidate remains very easy, the choice being largely reduced, because of the bipolarisation of American policy and moreover, the tasks suggested in the study of Fama et al. (2000) [12] included non-specific questions. According to Mayes et al., (1994) [21], it is necessary to use more specific questions in order to correctly evaluate remote memory. That is the reason all these measures were used in this Famous Events Task, specifically in pathologic groups having tiny difficulties [18, 19].

The difficulties, in terms of correlations (total events score and specific questions with the "recall" abilities of the MATTIS scale), possibly reflect an impairment in rebuilding memories. A positive correlation is also observed with the initiation score of the MATTIS, suggesting that the difficulties of rebuilding can be related to a dysfunction in accessing information because of a certain degree of frontal amnesia. The evolution of the memories for public events in non-demented PD patients suggests a dysfunction of total rebuilding rather than amnesia for the most recent facts as in Alzheimer disease [22]. Therefore, no temporal gradient is found in PD.

The pattern of results also revealed that the recall of FBM was impaired in PD, reflecting a temporal and contextual deficit and difficulties in accessing the memories [16]. In both populations, events associated with a FBM allow a more precise recall of them, which supports the episodic theories (traces are more specific and more easily retrievable within context). In the same way, results [23] are consistent with a continuum between the autobiographical and the public events memories. Also, the capacity to generate a FBM is correlated with the total capacity to retrieve the memory in its semantic component and the more general capacity to recall new information.

To conclude, the present study suggests an introduction into the expertise of PD of the evaluation of the semantic/episodic and temporal memory.
